# Increased Salivary microRNAs That Regulate DJ-1 Gene Expression as Potential Markers for Parkinson’s Disease

**DOI:** 10.3389/fnagi.2020.00210

**Published:** 2020-07-07

**Authors:** Yanmei Chen, Jingxue Zheng, Lufen Su, Fengxian Chen, Ruihan Zhu, Xiaochun Chen, Qinyong Ye

**Affiliations:** ^1^Department of Neurology, Fujian Medical University Union Hospital, Fuzhou, China; ^2^Institute of Neuroscience, Fujian Key Laboratory of Molecular Neurology, Fujian Medical University, Fuzhou, China

**Keywords:** Parkinson’s disease, DJ-1 protein, miR-874, miR-145-3p, saliva, biomarker

## Abstract

Small molecule RNAs (microRNAs) are a kind of endogenous, stable, and noncoding RNA molecule that can regulate the expression of target genes such as DJ-1 at the posttranscriptional level. This study aimed to detect the expression of salivary microRNAs and to discover their value as a salivary potential biomarker for Parkinson’s disease (PD). Through a case-control study, RT-qPCR technology was used to detect the expression of miR-874 and miR-145-3p in the saliva of 30 PD patients and 30 healthy volunteers. Then we compared the differences in the expression levels of salivary miR-874 and miR-145-3p between the PD group and the control group and analyzed the correlation between the expression of salivary miR-874 and miR-145-3p in terms of age, gender, disease condition, and disease course. We found that salivary miR-874 and miR-145-3p were both positively expressed in the PD group and control group, and their expression in the PD group was higher than that in the control group. The expression of salivary miRNA-874 and miR-145-3p had no clear correlation to age, gender, total RNA concentrations in saliva, the score of UPDRSII, UPDRSIII, olfactory test scale, MMSE, MoCA, Hohn–Yahr stage and disease course. In conclusion, in the PD group and the control group with positive expression, the expression levels of miR-874 and miR-145-3p in the PD group were higher than those in the control group. The detection of miR-874 and miR-145-3p expression in saliva can be used as an auxiliary biomarker for PD.

## Introduction

Parkinson’s disease (PD) is a common age-related neurodegenerative disease. The main pathological changes are the progressive degeneration of the dopaminergic (DA) neurons of the substantia nigra (SN) brain region and the abnormal deposition of the alpha-synuclein protein in the cytoplasm of the remaining dopaminergic neurons, which cannot be degraded *via* the ubiquitin-proteasome system (Damier et al., 1993; Zheng et al., 2010). Also, complex I activity and reduced glutathione content in the SN of PD patients are significantly reduced and oxidative stress is enhanced, which suggests that antioxidant dysfunction and oxidative stress may be associated with the onset and progression of PD (Hernandez et al., 2016).

The DJ-1 protein is a kind of multifunctional protective protein that participates in the different stages of cell growth and development, including transcriptional regulation, cell transformation, antioxidant stress reaction, molecular chaperoning, and protease and mitochondrial regulation. The decreased DJ-1 protein caused by the inactivated DJ-1 protein after oxidation was found in the midbrain SN of sporadic PD patients, which reveals that the DJ-1 gene participates in both familial and sporadic PD (Lev et al., 2006). However, the molecular mechanism of decreased DJ-1 protein is not yet clear.

In recent years, many studies have shown that small RNAs (microRNAs) act as a class of endogenous non-coding RNA molecules to regulate the expression of target genes at the posttranscriptional level, affecting cell growth and disease development. microRNAs are single-stranded RNA molecules with a length of approximately 20–25 nucleotides that widely exist in eukaryotes. Mature miRNA combines with the RNA-Induced Silencing Complex (RISC), which leads to the degradation or translation inhibition of target mRNA through complementary pairing with the 3′UTR of the target gene, thus exerting its effects on regulation of gene expression. microRNA and target gene mRNA does not have a one-to-one correspondence. One microRNA can regulate the expression of different mRNAs, while multiple microRNAs can regulate the expression of the same mRNA (Bartel, 2009). Researchers have found that most of the microRNAs are characterized by tissue specificity and temporal specificity (Wake et al., 2016) and can regulate their transcription (Saghazadeh and Rezaei, 2015). Their characteristics, temporal specificity, a significant change in expression level, and the specificity of the tissues or cells involved, suggest that microRNAs may participate in far-reaching and complex regulatory networks and play a decisive role in the growth development and behavior process. On the other hand, studies have shown that microRNAs play an important role in the pathogenesis of PD. For example, SNCA, LRRK2, and other genes are regulated by microRNAs. MiR-7 and miR-153 protect cells from oxidative stress-mediated cell death by inhibiting the expression of α-synuclein (Martinat et al., 2006). MiR-205 can inhibit the expression of the LRRK protein, and overexpressed miR-205 can prevent the neurite growth defects caused by the LRRK2 pathogenic mutation R1441G (Martinat et al., 2006). Therefore, it is speculated that the decrease in DJ-1 protein levels in sporadic and familial PD may be regulated protocol was as followsby specific microRNAs.

The comprehensive diagnoses of PD are based on the clinical manifestations, sufficient neurological assessment (UPDRS and Hohn–Yahr stage scale), imaging examination such as cranial MRI, SN ultrasound, and diagnostic treatment. The gold standard for diagnosis is tissue biopsy (Wake et al., 2016); however, it cannot be promoted clinically due to its obvious limitations (invasive operation and high risk). The DA neurons in the SN of the midbrain are degenerated and reduced 50–70% when PD patients show clinical manifestations. At this time, patients lose the opportunity for early treatment. Therefore, looking for objective and quantitative biomarkers can better assist early diagnosis and treatment of PD in the reduction of missed diagnosis and misdiagnosis. The specific microRNAs that may regulate the expression of DJ-1 is hsa-miRNA-874 and hsa-miR-145-3p, which are predicted by three miRNA target gene prediction software programs (http://www.microrna.org/, MicroCosm Targets, and TargetScanHuman 7.1).

Because microRNAs retain sufficient integrity and high concentration in body fluids including saliva (Dong et al., 2016), in this study, we determined whether a specific microRNA can be used as a quantitative PD-specific marker by detecting the expression levels of hsa-miRNA-874 and hsa-miR-145-3p in saliva, providing a new detection method for early diagnosis.

## Materials and Methods

### Participants

This study was performed in the Department of Neurology of Fujian Medical University Union Hospital, Fuzhou, China, and was approved by the Ethics Committees of Fujian Medical University Union Hospital. All individuals provided written informed consent. From October 2016 to March 2017, a total of 30 patients were recruited (ages 40–79, mean ± SD of 63.20 ± 10.17 years, 20 males and 10 females). The case inclusion criteria are based on the 2016 edition of the Chinese diagnostic criteria for PD (Postuma et al., 2016). The control group consisted of 30 healthy volunteers (ages 35–78, mean ± SD of 59.57 ± 12.83 years, 16 males and 14 females). There was no significant difference in age and gender among the two groups. The exclusion criteria for both groups were as follows: (1) the presence of diseases that may affect the secretion of saliva, such as upper respiratory tract infection, rhinitis, periodontitis, and periodontal abscess and other infectious diseases, or hemorrhagic wounds in the mouth; (2) the presence of connective tissue diseases, such as systemic lupus erythematosus (SLE), or Sjogren’s syndrome, and Behcet’s disease; and (3) the presence of severe disease of the heart or lungs, tumor lesions, neuropsychiatric disorders or intolerance to saliva collection.

### Saliva Collection

Saliva samples were collected at a fasting state in the morning. Saliva samples were centrifuged at 3,000 *g* for 15 min at 4°C and further centrifuged at 12,000 *g* for 10 min at 4°C (removing nonsoluble components in saliva). The supernatant was left after both centrifugations, and then bacteria were removed with a 0.22 mm microporous membrane filter. Then, all plasma samples were stored at −80°C.

### Test Procedures and Methods

#### Total RNA Extraction From Saliva

Three-hundred and fifty microliter of saliva was placed in a 1.5 ml EP tube, and 1 ml of Trizol Ls solution (American Invitrogen) was added for digestion and lysis. The sample was mixed with a pipette and placed at room temperature for 5 min for a complete breakdown. Then, chloroform was added at a ratio of 300 μl chloroform/1 ml Trizol Ls, and the mixture was shaken up and down for 20–30 times by hand and then incubated at room temperature for 12–15 min. After centrifugation at 12,000 *g* for 15 min at 4°C, the liquid in the tube was divided into three layers (upper aqueous phase, RNA; intermediate white film layer, DNA; bottom layer, protein). Approximately 700 μl of the upper aqueous phase was removed to another centrifuge tube while avoiding any removal of the white DNA film layer. Then, isopropanol was added at a ratio of 1 ml isopropanol/1 ml Trizol Ls and incubated at room temperature for 10 min. Following centrifugation at 12,000 *g* for 10 min at 4°C, the microRNA sank to the bottom of the tube and the supernatant was discarded. Then, 75% ethanol was added, which was prepared with anhydrous ethanol and DEPC water (Beyotime Institute of Biotechnology) at a ratio of 1 ml 75% ethanol/1 ml Trizol Ls, and the bottom of the tube was gently flicked to float the RNA precipitant. After centrifugation at 7,500 *g* for 5 min at 4°C, the supernatant was discarded. After air-drying at room temperature or vacuum-drying for 15–20 min, the microRNA samples were dissolved in 10 μl of DEPC water as final extracts. The total RNA content was analyzed by a nucleic acid-protein analyzer (American SpectraMax M3).

### Reverse Transcription-Quantitative Polymerase Chain Reaction

#### (RT-qPCR) Experiment

**Figure 1:**
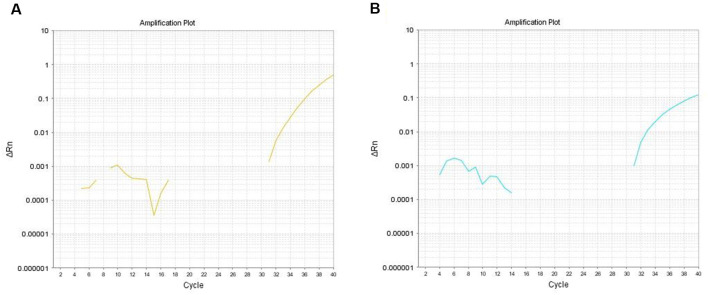
**(A)** The amplification curve of cDNA corresponding to miR-874 in the saliva of Parkinson’s disease (PD) patients. **(B)** The amplification curve of cDNA corresponding to miR-145-3p in the saliva of PD patients.

Total microRNA was reverse transcribed using the TaqMan^®^ microRNA Reverse Transcription Kit. All reagents were obtained from American Applied Biosystems, and all operations were conducted in strict accordance with the manufacturer’s protocol. The microRNA primer ID number were included as follows: hsa-miR-16 (internal reference) primer’s ID number was 000391, hsa-miR-874 primer’s ID number was 002268, and hsa-miR-145-3p primer’s ID number was 002149. The reverse transcription protocol was as follows: 30 min at 16°C and 30 min at 42°C for reverse transcription reaction and 5 min for enzyme inactivation reaction at 85°C. The obtained cDNA was used immediately for PCR, stored at −20°C for a week, or stored at −80°C for a long time. The qPCR experiment was performed using the TaqMan^®^ Assay reagent (20×). The qPCR protocol was as follows: 2 min for UNG activation at 50°C, 10 s for enzyme activation at 95°C, followed by 45 cycles of PCR template denaturation for 3 s at 95°C, and annealing and extending for 30 s at 60°C. The cDNA amplification curve was produced following RT-qPCR. The American ABI7500 Fluorescent Quantitative PCR instrument was used for detection. The primers used in reverse transcription and cDNA amplification were designed specifically by Applied Biosystems of the United States. The calculation of the relative miRNA expression levels was based on the 2^−ΔΔCt^ method. When the Ct value was greater than or equal to 36.5 or was “undetermined,” the detection result was considered negative if miRNA detection failed (Figure 1).

### Statistical Processing

In this study, SPSS Statistics 22.0 (IBM, Corp., Armonk, NY, USA) was used for statistical analyses. A *p*-value < 0.05 was accepted to be statistically significant in all cases. The data of normal distribution and variance homogeneity are expressed as the mean standard deviation ($\bar{x}$ ± s), while the data that do not conform to normal distribution or variance heterogeneity are expressed as the median (P25, P75). The categorical data were analyzed by *χ*^2^ test, the measurement data that conformed to a normal distribution and homogeneity of variance were analyzed by two independent-sample *t*-tests, and the measurement data that did not conform to a normal distribution or homogeneity of variance were analyzed by the Mann–Whitney *U*-test. Receiver operating characteristic (ROC) curves for the biomarkers were generated to evaluate their sensitivities and specificities in distinguishing PD patients from healthy controls. The “optimum” cut-off value for a ROC curve was the point associated with the maximal sum of sensitivity and specificity.

## Results

### Clinical Data of the PD Group and the Control Group

There was no statistically significant difference between the PD group and the control group in gender, age (Supplementary Table S1). There were 28 cases of positive expression of saliva miR-874, including 14 cases in the PD group and 14 cases in the control group and 35 cases of positive expression of saliva miR-145-3p, including 15 cases in the PD group and 20 cases in the control group.

### Positive Expression of miR-874 and miR-145-3p in Saliva in the PD Group and Control Group

Saliva was positive for miR-874 in 28 subjects (14 in the PD group and 14 in the control group) and was negative in 32 subjects (16 in the PD group and 16 in the control group). Statistical results are shown in Supplementary Table S1, with *χ*^2^ = 0.000 and *p* = 1.0 > 0.05, indicating that the positive expression of miR-874 in saliva was not significantly different between the two groups (Supplementary Table S2).

Saliva was positive for miR-145-3p in 35 subjects (15 in the PD group and 20 in the control group) and negative in 25 subjects (15 in the PD group and 10 in the control group). Statistical results are shown in Supplementary Table S2, with *χ*^2^ = 1.714 and *p* = 0.190 > 0.05, indicating that the positive expression of miR-145-3p in saliva was not significantly different between the two groups (Supplementary Table S3).

### Comparison of the Relative Expression of microRNAs in Saliva Between the PD Group and the Control Group With microRNAs Positive Expression

**Figure 2:**
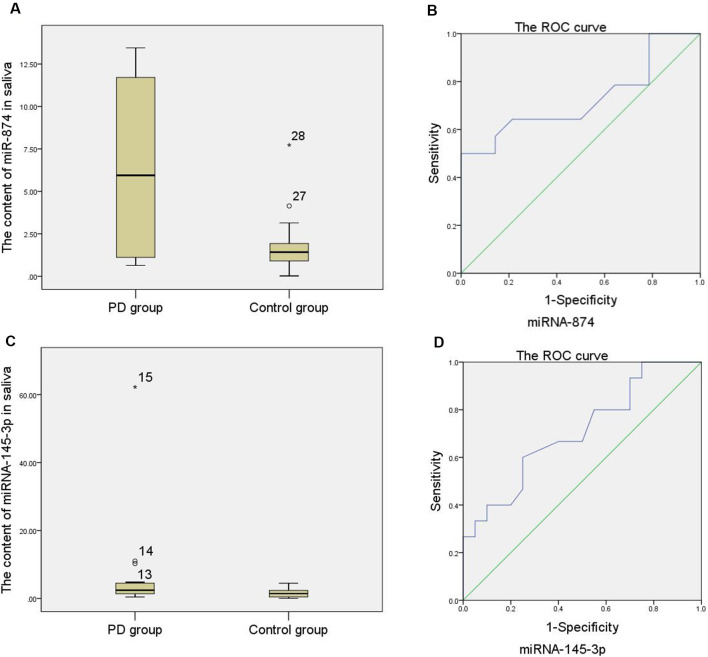
**(A)** Comparison of the relative expression of miR-874 in saliva between the PD group and the control group (Note: numbers 27 and 28 are the abnormal values of the relative expression of miR-874 in saliva in the control group). **(B)** Curve of the relative expression of miR-874 in saliva. **(C)** Comparison of the relative expression of miR-145-3p in saliva between the PD group and the control group (Note: numbers 13, 14, and 15 are abnormal values of the relative expression of miR-145-3p in saliva in the PD group). **(D)** Curve of the relative expression of miR-145-3p in saliva.

As shown in Supplementary Table S4 and Figures 2A,C, the relative expression of miR-874 and miR-145-3p in the saliva of the PD group was higher than that of the control group with a statistically significant difference (*p* < 0.05). An analysis of the ROC curve (Figures 2B,D) showed that when the area under the ROC curve is 0.727 and the relative expression of miR-874 in the saliva is 2.54 as the boundary value for distinguishing the PD group from the control group; the Jordan index is the largest, with a sensitivity of 64.3% and a specificity of 78.6%. When the area under the ROC curve is 0.707 and the relative expression of miR-145-3p in the saliva is 2.02 as the boundary value for distinguishing the PD group from the control group; the Jordan index is the largest, with a sensitivity of 60% and a specificity of 75%.

### Correlation Analysis of microRNA Expression in Saliva Between the PD Group and the Control Group

Spearman analysis was used to analyze the correlation between the relative expression of miR-874 and miR-145-3p in the saliva of PD group in terms of their age, sex, total RNA concentration in saliva, UPDRSII, UPDRSIII, Hohn–Yahr staging, olfactory score, MMSE, MoCA, and course of the disease. The relative expressions of miR-874 and miR-145-3p in the saliva of the control group were analyzed for correlation with their age, sex, and total RNA concentration in saliva (Supplementary Tables S5, S6). The results showed that there was no clear correlation between them.

## Discussion

Studies have shown that saliva is the end product of blood circulation, and microRNAs in saliva are stable and abundant (Bahn et al., 2015). The mature microRNAs in the cells with argonaute proteins in the cytoplasm (AGO) are assembled into a RISC complex (RNA-induced silencing complex, RISC). With the assistance of the RISC complex, microRNAs can induce the degradation or translation inhibition of target mRNA by complementary pairing with the 3′UTR of the target gene, thus playing a role in gene expression regulation. The intracellularly produced microRNAs are transported to the extracellular space through different transport mechanisms (Dong et al., 2016): (A) in the case of damaged tissue or tissue cell rupture, microRNAs are directly released into the blood; (B) covered with vesicles, microRNAs are secreted into the blood through exosomes, exfoliated vesicles and apoptotic bodies, whose outer membrane can prevent microRNAs from being degraded by RNA enzymes; and (C) the RISC complex plays a protective role. Exposed microRNAs will decompose immediately after entering the plasma while circulating microRNAs are stable under the same conditions. In other words, microRNA molecules in saliva can stably exist through the above protection mechanisms as reliable biological markers (Wake et al., 2016).

At present, the diagnosis of PD is mainly based on neurological examination and neuroimaging, both of which are subjective and lack sensitivity. At the same time, PD can be diagnosed by examination of the SN in midbrain through histopathological analysis, but brain tissue cannot be obtained from living people. Cerebrospinal fluid can reflect some pathophysiological changes occurring in the brain. However, cerebrospinal fluid can only be collected through a lumbar puncture. The inconvenience of this invasive collection process limits the use of cerebrospinal fluid. Blood is an ideal candidate for biomarker detection, but its sampling is also invasive. In contrast, the collection process for saliva samples is simple, non-invasive, and safe. Studies have confirmed the differential expression of microRNAs, hsa-miR-132–3p, hsa-miR-497–5p, and hsa-miR-133b, in the brain and hsa-miR-221–3p, hsa-miR-214–3p, and hsa-miR-29c-3p, in the blood (Schulz et al., 2019). By contrast, no significant signals were found in CSF. To date, there is still no study on whether the changes of microRNAs in saliva are correlated with PD. In this study, we detected the expression of miR-874 and miR-145-3p in the saliva of 60 subjects (30 PD patients and 30 healthy controls) and found that both of them were positively expressed in PD patients and healthy controls, and there was no significant difference between them (*p*-values were 1.0 and 0.19). The subgroup analysis was conducted in the PD group and the control group with microRNAs positive expression. We found that the relative expression levels of mir-874 and miR-145-3p in the saliva of the PD group were higher than those in the control group with a statistically significant difference (*p* < 0.05). The reported frequency of monogenetic forms of PD varies considerably across studies, depending on the genes tested, the racial and ethnic background of the cohort, and age at disease onset. As for DJ-1-associated PD, mutations of the mitochondrial DJ-1 gene (PARK7) are associated with autosomal recessive inheritance, age younger than 40 at the onset, slow progression, and good response to levodopa. Wild type DJ-1 is thought to be neuroprotective against oxidative stress. In this study, given that both of miR-874 and miR-145-3p could be detected from PD patients and healthy controls with no significant difference, we hypothesized that these two microRNAs may be normally expressed in the human body to maintain the normal DJ-1 physiological function. The expression level of them in the control group is considered within the normal range. When the expression level is abnormally increased, it can lead to decreased expression of DJ-1 and mitochondrial dysfunction. Theoretically, the greater increase of expression of miR-874 and miR-145-3p will lead to the greater inhibition of DJ-1 protein expression, resulting in the lower concentration of DJ-1 protein and the more severe pathological condition. However, the results of this study showed that the expression of salivary miRNA-874 and miR-145-3p had no clear correlation to age, gender, total RNA concentrations in saliva, the score of UPDRSII, UPDRSIII, olfactory test scale, MMSE, MoCA, Hohn–Yahr stage and disease course. The reasons may be as follows: (1) microRNAs function by complementary pairing with the 3′UTR (3′ untranslated region) of the target mRNA. If they are completely matched, microRNAs directly degrade target mRNA; and if they are not completely matched, the microRNA-mediated degradation of mRNA is abolished. Also, due to existing nucleotide variations at 3′UTR terminal of target mRNA between different individuals, the inhibition of DJ-1 protein expression will be various. (2) The patients in this study were not DJ-1 mutation carriers, their pathogenesis of PD should be due to the interplay of multiple factors besides the decrease of DJ-1 protein. (3) The sample size was too small.

To further evaluate the potential for mir-874 and miR-145-3p in saliva to aid in the diagnosis of PD, ROC analysis was performed to characterize their sensitivity and specificity. The AUC for saliva miR-874 was 0.727, when the cut-off value was 2.54, with a sensitivity of 64.3% and a specificity of 78.6%. miR-145-3p generated a similar AUC result (0.707) with a sensitivity of 60% and a specificity of 75% at a cut-off value of 2.02. Therefore, it can be concluded that the high specific expression level of miR-874 and miR-145-3p in saliva can be used not only as a quantitative biological marker for PD diagnosis but also as a meaningful screening indicator.

## Conclusion

MiR-874 and miR-145-3p regulated DJ-1 gene expression in saliva may serve as potential biomarkers for PD. Further studies should be conducted with larger patient cohorts to corroborate the significance of these findings and the relationship of these biomarkers and disease.

## Data Availability Statement

The datasets generated for this study are available on request to the corresponding author.

## Ethics Statement

The studies involving human participants were reviewed and approved by Ethics committee of the Affiliated Union Hospital of Fujian Medical University. The patients/participants provided their written informed consent to participate in this study.

## Author Contributions

YC was responsible for the conception and design of the present study, execution of the experimental work, and wrote the first draft of the manuscript, review and critique of the manuscript. JZ undertook study design, execution of statistical analysis, execution of experimental work, and the review and critique of the manuscript. LS organized the research project and reviewed and critiqued the manuscript. FC and RZ organized the research project and reviewed and critiqued the statistical analysis and the manuscript. XC guided the design of the study protocol. QY was responsible for the conception of experiments, execution of experimental work, design and execution of statistical analysis, and the review and critique of the manuscript.

## Conflict of Interest

The authors declare that the research was conducted in the absence of any commercial or financial relationships that could be construed as a potential conflict of interest.
