# Supplementary material for: Increased Salivary microRNAs That Regulate DJ-1 Gene Expression as Potential Markers for Parkinson’s Disease
Source: Front Aging Neurosci. 2020 Jul 7;12:210. doi: 10.3389/fnagi.2020.00210 (PMC7360355; doi:10.3389/fnagi.2020.00210)
Supplement: Supplementary file 1 [file Table_1.docx]

Table 1 General clinical data of PD group and control group

| Clinical project | PD group | Control group | P value |
| --- | --- | --- | --- |
| Gender (male/female) | 20/10 | 15/15 | 0.190 |
| Age (year) | 63.2±10.17 | 59.57±12.83 | 0.229 |
| UPDRSⅡ | 9（7,14） | - | - |
| UPDRSⅢ | 24.43±9.55 | - | - |
| Hohn-Yahr stage | 2.0（1.38,2.5） | - | - |
| The sense of smell score | 4.5（1,7） | - | - |
| MMSE | 28（25.75,29） | - | - |
| MoCA | 21.30±4.07 | - | - |
| Hohn-Yahr stage | 30（12,51） | - | - |
