# Supplementary material for: Increased Salivary microRNAs That Regulate DJ-1 Gene Expression as Potential Markers for Parkinson’s Disease
Source: Front Aging Neurosci. 2020 Jul 7;12:210. doi: 10.3389/fnagi.2020.00210 (PMC7360355; doi:10.3389/fnagi.2020.00210)
Supplement: Supplementary file 3 [file Table_3.DOCX]

Table 3 Positive expression of miR-145-3p in saliva in the PD group and control group

| Clinical project | PD group | Control group | Total |
| --- | --- | --- | --- |
| Positive (number of cases) | 15 | 20 | 35 |
| Negative (number of cases) | 15 | 10 | 25 |
| Total (number of cases) | 30 | 30 | 60 |
