# Supplementary material for: Increased Salivary microRNAs That Regulate DJ-1 Gene Expression as Potential Markers for Parkinson’s Disease
Source: Front Aging Neurosci. 2020 Jul 7;12:210. doi: 10.3389/fnagi.2020.00210 (PMC7360355; doi:10.3389/fnagi.2020.00210)
Supplement: Supplementary file 4 [file Table_4.DOCX]

Table 4 Comparison of miRNA-874 and miRNA-145-3p expression in saliva between the PD group and control group

| microRNA | PD group | Control group（n=14） | p value |
| --- | --- | --- | --- |
| miR-874 | 5.95（1.03,11.92）（n=14） | 1.42（0.80,2.23）  （n=14） | 0.041* |
| miR-145-3p | 2.39（1.38,4.79）  （n=15） | 1.43（0.36,2.55）  （n=20） | 0.039* |

* Mann-Whitney U test, *p* <0.05
