# Supplementary material for: Increased Salivary microRNAs That Regulate DJ-1 Gene Expression as Potential Markers for Parkinson’s Disease
Source: Front Aging Neurosci. 2020 Jul 7;12:210. doi: 10.3389/fnagi.2020.00210 (PMC7360355; doi:10.3389/fnagi.2020.00210)
Supplement: Supplementary file 5 [file Table_5.DOCX]

Table 5 Correlation analysis of the relative expression of miRNA-874 in saliva between PD group and control group

| Bivariate of the study | r value | P values |
| --- | --- | --- |
| Gender -miRNA content | 0.000 | 1.000 |
| Age -miRNA content | -0.094 | 0.75 |
| Total RNA concentration in saliva -miRNA content | 0.067 | 0.819 |
| UPDRSⅡ-  miRNA content | 0.100 | 0.734 |
| UPDRSⅢ-  miRNA content | -0.207 | 0.477 |
| Hohn-Yahr stage-  miRNA content | 0.109 | 0.711 |
| The sense of smell score -miRNA content | 0.319 | 0.267 |
| MMSE-  miRNA content | 0.385 | 0.174 |
| MoCA-  miRNA content | 0.169 | 0.563 |
| Course-miRNA content | 0.139 | 0.635 |
