# Supplementary material for: Increased Salivary microRNAs That Regulate DJ-1 Gene Expression as Potential Markers for Parkinson’s Disease
Source: Front Aging Neurosci. 2020 Jul 7;12:210. doi: 10.3389/fnagi.2020.00210 (PMC7360355; doi:10.3389/fnagi.2020.00210)
Supplement: Supplementary file 6 [file Table_6.DOCX]

Table 6 Correlation analysis of the relative expression of miRNA-145-3p in saliva of PD group and control group

| Bivariate of the study | r value | P values |
| --- | --- | --- |
| Gender -miRNA content | -0.425 | 0.115 |
| Age -miRNA content | -0.213 | 0.447 |
| Total RNA concentration in saliva -miRNA content | 0.038 | 0.894 |
| UPDRSⅡ-  miRNA content | 0.185 | 0.508 |
| UPDRSⅢ-  miRNA content | -0.051 | 0.857 |
| Hohn-Yahr stage-  miRNA content | 0.283 | 0.307 |
| The sense of smell score -miRNA content | -0.033 | 0.906 |
| MMSE-  miRNA content | -0.308 | 0.264 |
| MoCA-  miRNA content | -0.053 | 0.851 |
| Course-miRNA content | -0.096 | 0.735 |
